# Effects of Traditional Kampo Drugs and Their Constituent Crude Drugs on Influenza Virus Replication *In Vitro*: Suppression of Viral Protein Synthesis by Glycyrrhizae Radix

**DOI:** 10.1155/2019/3230906

**Published:** 2019-12-03

**Authors:** Toshihito Nomura, Masaya Fukushi, Kosuke Oda, Akifumi Higashiura, Takashi Irie, Takemasa Sakaguchi

**Affiliations:** Department of Virology, Institute of Biomedical and Health Sciences, Hiroshima University, Hiroshima, Japan

## Abstract

An influenza virus epidemic is an important issue in public hygiene, and continuous development on an effective drug is required. Kampo medicine is a traditional medicine that is used clinically for treatment of various diseases in Japan and other East Asian countries. We evaluated the effects of the Kampo drugs maoto, kakkonto, senkyuchachosan, jinkokato, and bakumondoto, which are prescribed for treatment of respiratory symptoms including symptoms caused by influenza, on influenza virus replication in cultured cells. Culture media of influenza virus-infected MDCK(+) cells were tested for hemagglutination and infectivity at 24 h after the addition of Kampo drugs at various concentrations, and four of the five Kampo drugs were found to inhibit virus release to the culture media. These drugs inactivated virus infectivity not by acting on virus particles but by acting on virus-infected cells. In addition, when six crude drugs (*Atractylodis lanceae rhizome*, *Citri unshiu pericarpium*, *Cnidii rhizome*, *Glycyrrhizae radix*, *Rehmanniae radix*, and *Saposhnikoviae radix*) that constitute the effective Kampo drugs were examined, the strongest activity was found for *Glycyrrhizae radix* (IC_50_ = 0.27 mg/ml), which selectively suppressed viral protein synthesis. Since *Glycyrrhizae radix* is contained in many Kampo drugs, it may give anti-influenza virus activity to a broad range of Kampo drugs.

## 1. Introduction

Influenza viruses, which belong to the family *Orthomyxoviridae*, are enveloped viruses with segmented, single-stranded, negative-sense RNA genomes [1]. In seasonal influenza epidemics, mortality is a particular concern in the elderly [2], and some children may require inpatient care [3]. In addition, a new type of influenza virus may emerge and cause a pandemic. Several antiviral drugs including neuraminidase inhibitors have been developed for treatment of influenza. However, there are problems such as high cost and the possibility of appearance of drug-resistant virus variants. The development of new anti-influenza drugs and the establishment of novel therapeutic measures are needed.

Kampo medicine is a medical system that has been systematically organized on the basis of oriental medicine and distinctive drugs. Kampo medicine originated in ancient Chinese medicine and was introduced into Japan in the fifth or sixth century. In the seventeenth century, Kampo medicine underwent major developments that resulted in the style that is now practiced [4]. Kampo drugs used in Kampo medicine are essentially mixtures of extracts of various plants, fungi, minerals, and insects (called crude drugs). Indications are given for each Kampo drug, and the Japanese Ministry of Health, Labour, and Welfare has approved the use of Kampo drugs. Kampo drugs, which are sold at pharmacies or prescribed by physicians, are currently being used in Japan and other East Asian countries.

Maoto is often prescribed for influenza. Maoto was shown to act as an antipyretic in children [5] and to be more effective than oseltamivir in children younger than 5 years of age [6]. Maoto has also been reported to inhibit virus replication in cultured cells [7]. However, reports on the effects of other Kampo drugs on virus replication *in vitro* are limited.

In the present study, we compared the effects of the Kampo drugs maoto, kakkonto, senkyuchachosan, jinkokato, and bakumondoto, which are prescribed for respiratory symptoms including symptoms caused by influenza and common cold, on influenza virus replication *in vitro*. A similar study was also carried out on the crude drugs included in these Kampo drugs, and strong activity of *Glycyrrhizae radix* was found.

## 2. Materials and Methods

### 2.1. Cells, Viruses, and Antibodies

MDCK(+) cells, canine kidney-derived cells described in [8], were propagated in Dulbecco's modified Eagle's minimum essential medium (DMEM, Invitrogen) supplement with 10% fetal calf serum (FCS; Biosera, Kansas City, MO, U.S.A.), penicillin G (100 units/ml, Meiji Seika Pharma, Tokyo, Japan), and streptomycin (100 *µ*g/ml, Meiji Seika Pharma). Human influenza virus strain A/Udorn/72 (H3N2) was propagated in 10-day-old embryonated chicken eggs as described previously [9], and its infectivity was measured by calculating the 50% tissue culture infectious dose (TCID_50_) in MDCK(+) cells in a 96-well plate as described previously [10]. Antiserum against influenza virus was prepared by immunizing a rabbit with purified A/Udorn/72 (H3N2) virions [9].

### 2.2. Reagents

Kampo drugs, maoto, kakkonto, jinkokato, senkyuchachosan, and bakumondoto (Table 1), were purchased from Tsumura & Co. (Tokyo, Japan). Constituents of the Kampo drugs, crude drugs, and the ratios of crude drugs included in Kampo drugs are posted at the web site of Tsumura & Co. (summarized in Table 2). Crude drugs, *Glycyrrhizae radix*, *Atractylodis lanceae rhizome*, *Rehmanniae radix*, *Citri unshiu pericarpium*, *Cnidii rhizome*, and *Saposhnikoviae radix* (Table 2), were also purchased from Tsumura & Co.

Kampo drugs were dissolved at concentrations of 2.5 or 3 mg/ml in DMEM supplemented with penicillin G and streptomycin at 50°C for 1 h, clarified by low-speed centrifugation following the manufacturer's instructions and sterilized with a 0.22-*µ*m filter. One milliliter of the filtrate was lyophilized, and the actual weight of the solute was measured. Crude drugs were similarly dissolved in DMEM and filtrated as Kampo drugs. Since almost no insoluble material was found after the low-speed centrifugation, the weight of the initial powder was used for the weight of the solute.

### 2.3. Cytotoxicity Assay

MDCK(+) cells were maintained in DMEM containing a designated concentration of a reagent for 24 h, and lactate dehydrogenase (LDH) in the medium was measured by a colorimetric method using the Cytotoxicity LDH Assay Kit-WST (DOJINDO Laboratories, Kumamoto, Japan) and the plate reader TriStar LB 941 (Bertohold Technologies, Wildbad, Germany) with absorbance at 485 nm.

### 2.4. Assay for Inactivation of Virus Particles

For Kampo drugs, 90 *µ*l of a drug at a concentration ranging from 20.6 to 24.5 mg/ml was mixed with 10 *µ*l of a virus solution of 6.3 × 10^8^ TCID_50_/ml and incubated for 3 min at room temperature. The mixture was then 10-fold serially diluted with DMEM, and infectivity in the solution was measured by the TCID_50_ method. For crude drugs, a reagent at a concentration of 12.5 mg/ml was used. Ethanol (70%) was used for an inactivating control.

### 2.5. Investigation of Influenza Virus Replication *In Vitro*

MDCK(+) cells were infected with the virus at an input multiplicity of infection (m.o.i.) of 5 or 0.2. After 1 h adsorption, the virus inoculum was removed and the cells were further incubated in DMEM containing a Kampo drug or a crude drug at different concentrations and 30 *µ*g/ml of trypsin (Merck, Darmstadt, Germany). After 24 h, the medium was harvested and processed for a hemagglutinin (HA) test with chicken erythrocytes and for virus infectivity measurement by the TCID_50_ method. The 50% inhibitory concentration (IC_50_) of a reagent was calculated by using the media from cells infected at an input m.o.i. of 0.2. Logarithms of the infectivity titers in the medium and concentrations of a reagent were plotted in a graph, and a linear approximate line was drawn for the calculation.

### 2.6. Evaluation of Protein Synthesis Inhibition by Crude Drugs

MDCK(+) cells were infected with the virus at an input m.o.i. of 5, and after 1 h of virus adsorption, the inoculum was replaced with DMEM containing 3.2, 6.3, or 12.5 mg/ml of *Glycyrrhizae radix* or *Cnidii rhizome*. At 7 h after infection, the cells were incubated for 30 min in methionine- and cysteine-free DMEM (Invitrogen, Thermo Fisher Scientific, USA) and then labeled with EXPRESS Protein Labeling Mix, [^35^S] (PerkinElmer, Inc., Waltham, MA, USA) for 20 min. The cells were lysed in a radioimmunoprecipitation assay (RIPA) buffer (10 mM Tris–HCl, pH 7.4, 1% Triton X-100, 1% sodium deoxycholate, 0.1% sodium dodecyl sulfate (SDS), 150 mM NaCl, and Complete proteinase inhibitor cocktail (Roche Diagnostics)) as described previously [11]. Proteins were immunoprecipitated by using anti-influenza virus rabbit serum and protein A-conjugated Sepharose beads (Pharmacia, Sweden) and analyzed by 10% SDS-polyacrylamide gel electrophoresis (PAGE). Radioactivity was analyzed by using a Fujix BAS 2000 image analyzer (Fuji Medical Systems, Inc., Tokyo, Japan).

## 3. Results

### 3.1. Effects of Kampo Drugs with Indications for Respiratory Symptoms on Influenza Virus Replication

The Kampo drugs used in this study are listed in Table 1. Chinese characters, Tsumura number, and English names (Japanese reading and Chinese reading) are shown, and English names (Japanese reading) are used hereafter throughout this paper. Their indications include common cold, influenza, and respiratory symptoms including cough and sputum, and only maoto is indicated for “influenza.” We investigated whether these drugs inhibited virus growth in cultured cells.

**Figure 1:**
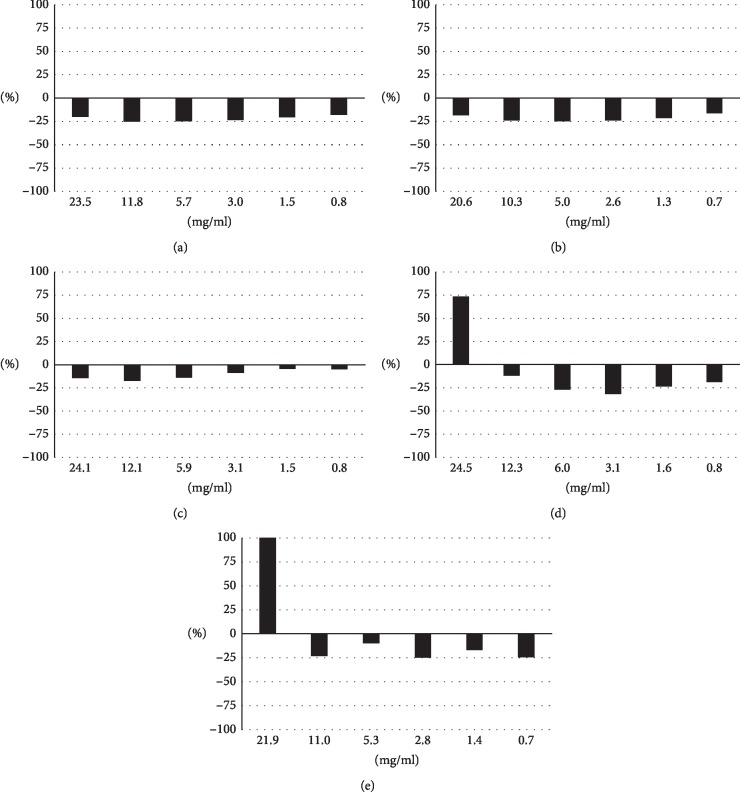
Cytotoxicities of Kampo drugs. MDCK(+) cells were incubated in DMEMs supplemented with the designated concentrations of a Kampo drug for 24 h. LDH values in the media were then measured to evaluate cytotoxicity. The LDH value from detergent-treated cells was set at 100%, and relative concentrations are shown in the graph. (a) Maoto. (b) Kakkonto. (c) Jiinkokato. (d) Senkyuchachosan. (e) Bakumondoto.

The cytotoxicity of the Kampo drugs against MDCK(+) cells used in infection experiments was investigated. A Kampo drug was added to the culture media at various concentrations indicated in the figure, and cytotoxicity assays were performed to measure the levels of the deviating enzyme LDH in the media after 24 h (Figure 1). High LDH values were obtained at the highest concentration of 24.5 mg/ml for senkyuchachosan and at the highest concentration of 21.9 mg/ml for bakumondoto, but there was no increase in LDH in other conditions, indicating almost no cytotoxicity of the drugs.

**Figure 2:**
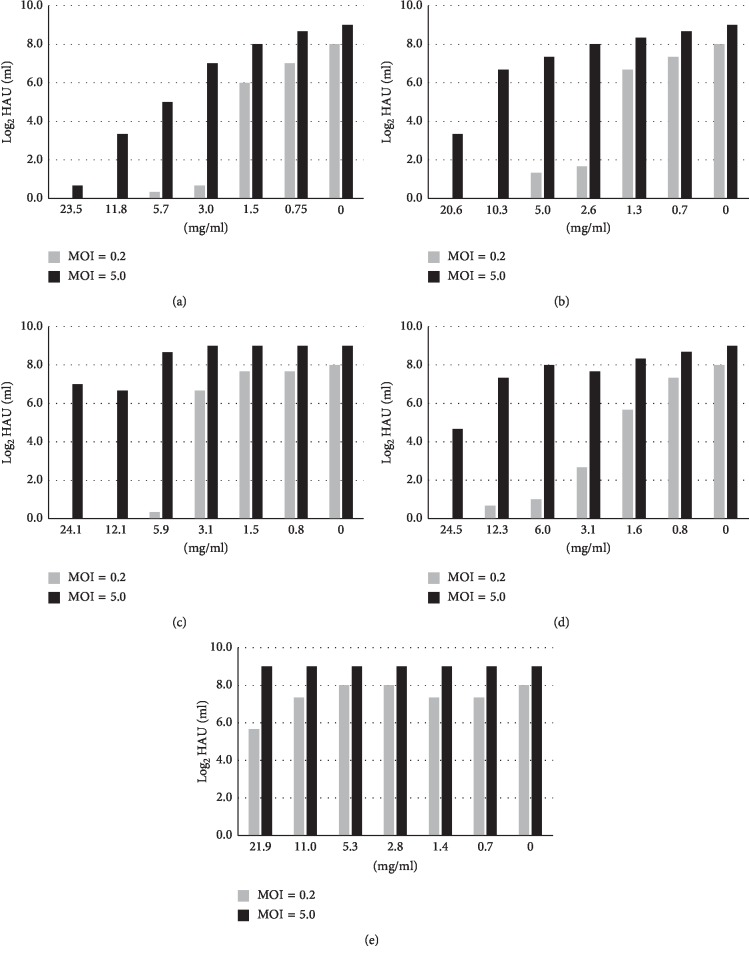
Virus release from Kampo drug-treated, virus-infected cells. MDCK(+) cells were infected with influenza virus at an input m.o.i. of 0.2 or 5 and incubated in the media containing the designated concentrations of a Kampo drug. After 24 h, HA activities in the media were measured. Gray bars, m.o.i. = 0.2; black bars, m.o.i. = 5. (a) Maoto. (b) Kakkonto. (c) Jiinkokato. (d) Senkyuchachosan. (e) Bakumondoto.

MDCK(+) cells were infected with influenza virus at an m.o.i. of 0.2 or 5, and then various concentrations of a Kampo drug were added to the culture media. After 24 h, the HA titers of the culture media were measured (Figure 2). In the case of an m.o.i. of 5, a condition in which all cells are infected, maoto had inhibitory effects depending on its concentrations. Kakkonto and senkyuchachosan also had inhibitory effects at high concentrations. On the other hand, at an m.o.i. of 0.2, a condition in which some of the cells are infected first and then the virus spreads, all of the Kampo drugs except bakumondoto suppressed HA titers at around or above the level of 3 mg/ml (Figure 2).

**Figure 3:**
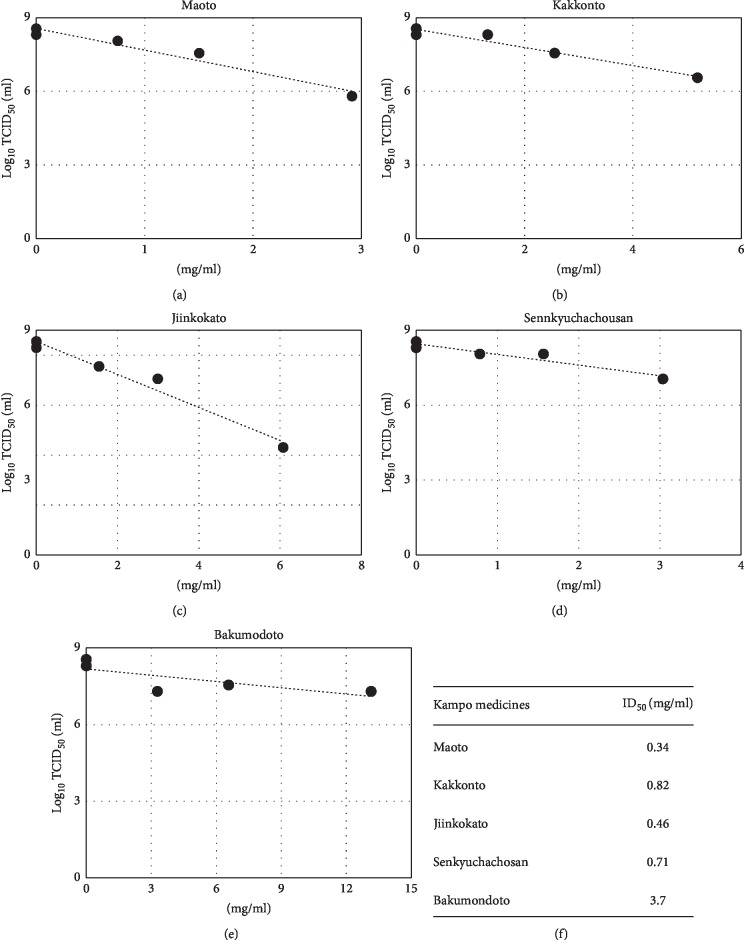
Measurement of infectious titers and calculation of IC_50_. Virus infectivity in the medium prepared as described in the legend of Figure 2 at an m.o.i. of 0.2 was measured. The logarithms of infectious titers and Kampo drug concentrations were plotted, and IC_50_ values were calculated on the basis of a linear approximated line.

Viral infectivity titers were measured at various concentrations, and IC_50_ values were calculated to be 0.34, 0.82, 0.46, 0.71, and 3.7 mg/ml for kakkonto, maoto, jiinkokato, senkyuchachosan, and bakumondoto, respectively, under the condition of an m.o.i. of 0.2 (Figure 3).

These results suggest that maoto inhibited the replication stages of influenza virus in the cells such as protein synthesis and intracellular maturation. On the other hand, the results suggest that jinkokato inhibited stages of virus spread such as budding of progeny viruses from virus-infected cells and further infection to surrounding cells. Kakkonto and senkyuchachosan may have inhibited multiple stages both in the cells and in virus spread.

**Figure 4:**
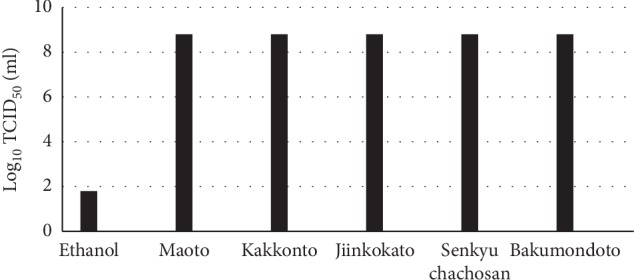
Effects of a Kampo drug on virus infectivity. Virus (10 *µ*l) was incubated with a Kampo drug at the highest concentration shown in Figure 2 (90 *µ*l) for 3 min at room temperature. Then, infectivity of the mixture was measured.

Each Kampo drug at the highest concentrations shown in Figure 2 was mixed with the virus, and infectivity was measured (Figure 4). The Kampo drugs did not significantly reduce the infectivity, indicating that the Kampo drugs did not inactivate virus infectivity. This indicates that the Kampo drugs act on virus-infected cells, not virus particles.

### 3.2. Investigation of Crude Drugs as Constituents of Kampo Drugs

Kampo drugs are mixtures of multiple crude drugs, each of which is derived mainly from roots and leaves of plants. Table 2 shows the compositions of crude drugs contained in the Kampo drugs that were tested for their ability to inhibit influenza virus replication. Maoto and kakkonto, both of which inhibited virus replication (Figures 2 and 3), contain *Ephedrae herba*. Since there are reports on the antiviral activity of *Ephedrae herba* [12, 13], its antiviral activity was not analyzed in this study.

Of the drugs listed in Table 2, we focused on crude drugs contained in jiinkokato and senkyuchachosan, and we were able to obtain six crude drugs (Table 2, italicized). Among those crude drugs, *Glycyrrhizae radix* was found in all of the five Kampo drugs listed in Table 1 (Table 2). The anti-influenza virus activities of the six crude drugs were examined.

**Figure 5:**
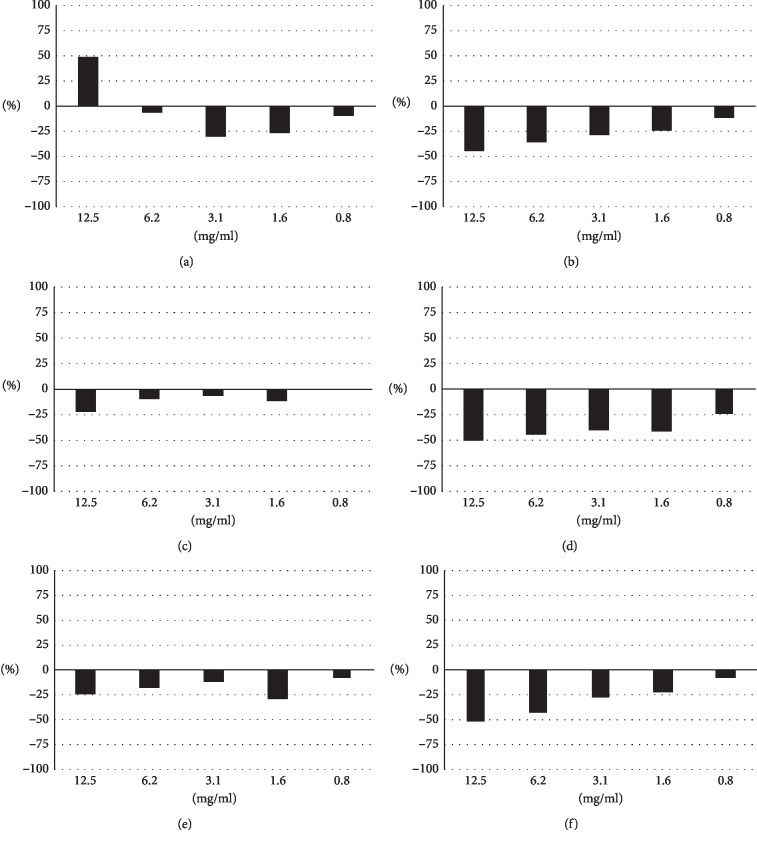
Cytotoxicities of crude drugs. MDCK(+) cells were incubated in DMEMs supplemented with the designated concentrations of a crude drug for 24 h. LDH values in the media were then measured to evaluate cytotoxicity. The LDH value from detergent-treated cells was set at 100%, and relative concentrations are shown in the graph. (a) *Glycyrrhizae radix*. (b) *Atractylodis lanceae rhizoma*. (c) *Rehmanniae radix*. (d) *Citri unshiu pericarpium*. (e) *Cnidii rhizoma*. (f) *Saposhnikoviae radix*.

The cytotoxicities of the crude drugs for MDCK(+) cells were investigated by LDH assays. The assays showed some cytotoxicity of *Glycyrrhizae radix* at its highest concentration (12.5 mg/ml), but the other crude drugs showed no cytotoxicity at any concentration (Figure 5).

**Figure 6:**
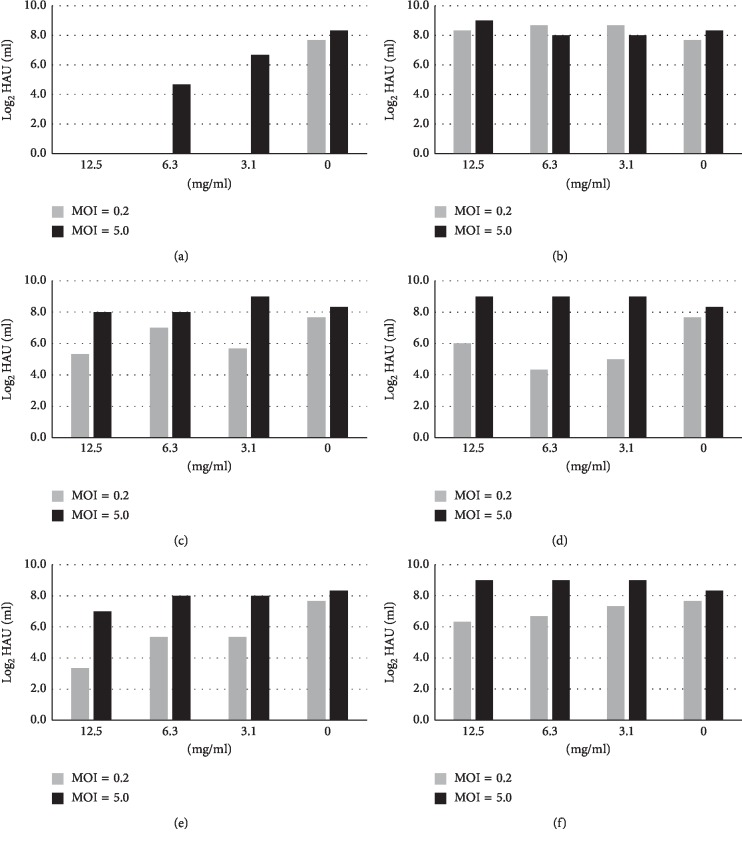
Virus release from crude drug-treated, virus-infected cells. MDCK(+) cells were infected with influenza virus at an input m.o.i. of 0.2 or 5 and incubated in the media containing the designated concentrations of a crude drug. After 24 h, HA activities in the media were measured. Gray bars, m.o.i. = 0.2; black bars, m.o.i. = 5. (a) *Glycyrrhizae radix*. (b) *Atractylodis lanceae rhizoma*. (c) *Rehmanniae radix*. (d) *Citri unshiu pericarpium*. (e) *Cnidii rhizoma*. (f) *Saposhnikoviae radix*.

**Figure 7:**
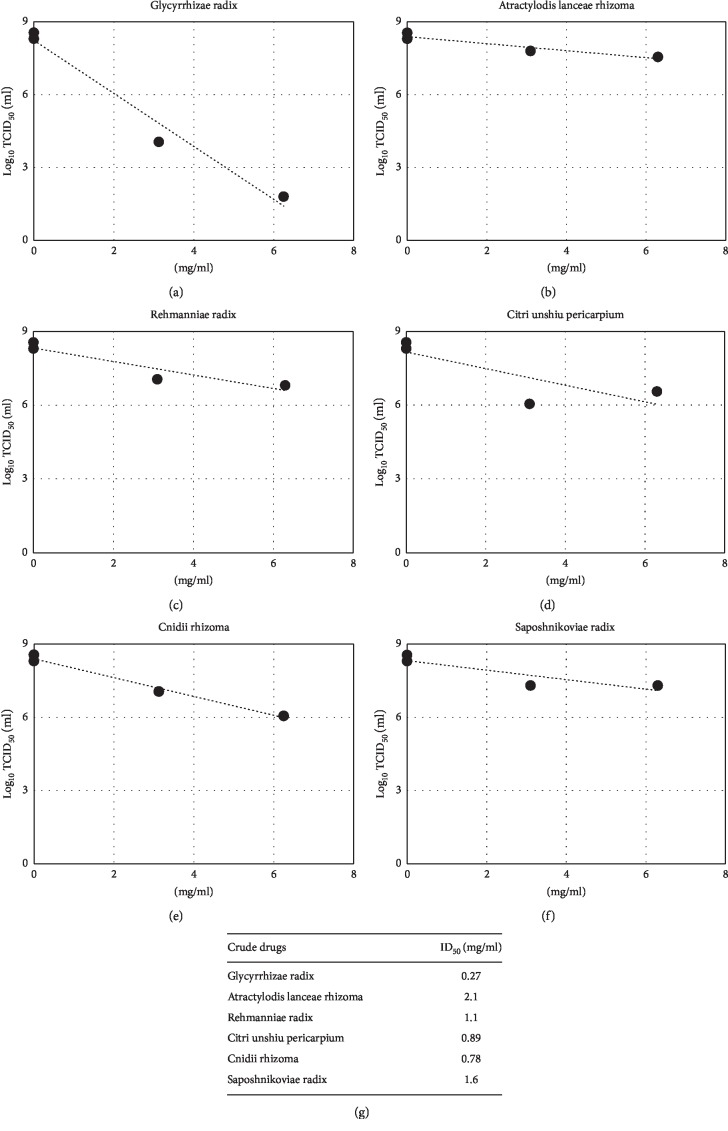
Measurement of infectious titers and calculation of IC_50_. Virus infectivity in the medium prepared as described in the legend of Figure 6 at an m.o.i. of 0.2 was measured. The logarithms of infectious titers and crude drug concentrations were plotted, and IC_50_ values were calculated on the basis of a linear approximated line.

As shown in Figure 2, when MDCK(+) cells were infected with influenza virus at an m.o.i. of 5, strong inhibitory activity of *Glycyrrhizae radix* was observed. At 6.3 mg/ml, a concentration at which no cytotoxicity was observed, *Glycyrrhizae radix* inhibited viral growth. Infection at an m.o.i. of 0.2 also resulted in inhibition of virus replication by *Glycyrrhizae radix* and by *Cnidii rhizome* (Figure 6). When IC_50_ was assessed in virus infection at an m.o.i. of 0.2, *Glycyrrhizae radix* showed the lowest IC_50_ value (0.27 mg/ml), followed by *Cnidii rhizoma* (0.78), *Citri unshiu pericarpium* (0.89), *Rehmanniae radix* (1.1), and *Atractylois lanceae rhizome* (2.1) (Figure 7).

**Figure 8:**
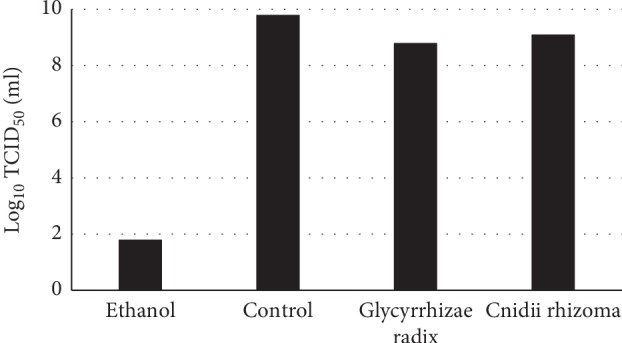
Effect of a crude drug on virus infectivity. Virus (10 *µ*l) was incubated with a crude drug at the highest concentration shown in Figure 6 (90 *µ*l) for 3 min at room temperature. Then, infectivity of the mixture was measured.

For *Glycyrrhizae radix*, which showed particularly strong anti-influenza activity, and *Cnidii rhizoma*, inactivation of virus infectivity was examined by mixing the virus with the crude drug at a concentration of 12.5 mg/ml. The virus titers were not reduced, indicating that these crude drugs did not inactivate virus particles (Figure 8).

### 3.3. Inhibition of Protein Synthesis by Crude Drugs

**Figure 9:**
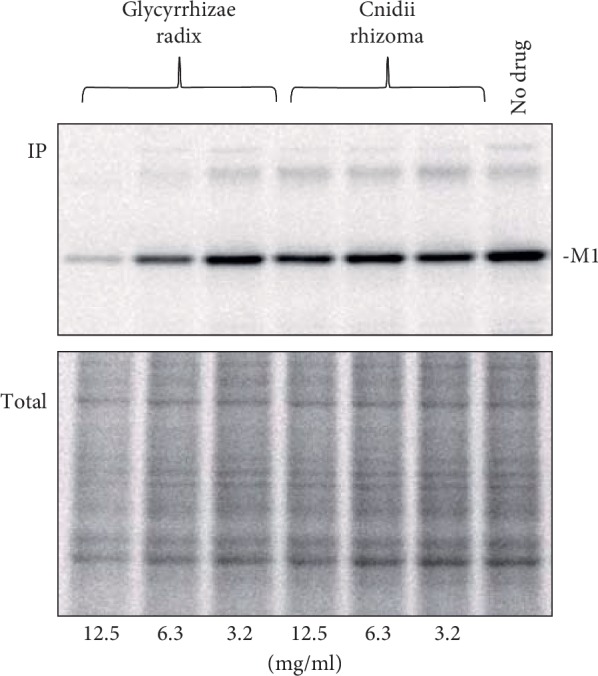
Inhibition of protein synthesis by crude drugs. MDCK(+) cells were infected with influenza virus at an input m.o.i. of 5 and maintained in DMEM containing 3.2, 6.3, or 12.5 mg/ml of *Glycyrrhizae radix* or *Cnidii rhizome*. At 7 h after infection, the cells were pulse-labeled with EXPRESS Protein Labeling Mix, [^35^S] for 20 min. Proteins were immunoprecipitated by using anti-influenza virus rabbit serum and analyzed by 10% SDS-PAGE. Radioactivity was analyzed by using a Fujix BAS 2000 image analyzer. IP: immunoprecipitated with an anti-influenza virus antibody. Total: cell lysates before immunoprecipitation were analyzed by SDS-PAGE and a part of the image corresponding to the region of M1 protein roughly ranging from 25 to 60 kDa is shown.

Inhibition of viral protein synthesis by *Glycyrrhizae radix* and *Cnidii rhizome* was also examined. MDCK(+) cells were infected with influenza virus at an m.o.i. of 5 and cultured in the presence of the crude drugs at various concentrations. At 7 h after infection, proteins were pulse-labeled with ^35^S-Cys and -Met for 20 minutes and immunoprecipitated with an anti-influenza virus antibody. *Glycyrrhizae radix* specifically inhibited viral protein synthesis without affecting overall cellular protein synthesis (Figure 9). Only the M1 protein is shown in the figure, but similar results were obtained for other viral proteins (data not shown). On the other hand, *Cnidii rhizoma* did not inhibit viral protein synthesis.

## 4. Discussion

The emergence of drug-resistant viruses with the development of anti-influenza virus drugs is becoming a problem. The 2009 Pandemic virus was resistant to amantadine, an M2 ion channel blocker, from the beginning, and almost all of the current seasonal influenza viruses and avian H5N1 viruses have acquired resistance to amantadine. Viruses that are resistant to neuraminidase inhibitors have also emerged [14]. A particular case is baloxavir marboxil, a recently developed drug that was prescribed the most frequently among anti-influenza drugs in the 2018-2019 season in Japan, and emergence of baloxavir marboxil-resistant point mutants has been reported [15]. Due to the emergence of drug-resistant viruses, a new anti-influenza virus drug must be developed.

In the present study, five Kampo drugs that are prescribed for patients with respiratory symptoms were examined for their effects on influenza virus replication in cultured cells. Four of the five Kampo drugs suppressed virus replication. Among the Kampo drugs, the anti-influenza virus activity of maoto was particularly potent and maoto suppressed replication of the virus at an m.o.i. of 5, suggesting that it can inhibit the growth of a virus in infected cells. Kakkonto also weakly inhibited the growth of influenza virus at an m.o.i. of 5. At infection with an m.o.i. of 0.2, virus growth was markedly inhibited by jiinkokato and senkyuchachosan, indicating that the production of progeny virions or the process of infecting nearby cells was inhibited. On the other hand, these drugs did not inactivate virus particles.

Maoto has been shown to be effective for reducing fever in children infected with influenza viruses [5, 6]. Infection experiments with an influenza virus in cultured cells have also shown that maoto inhibits virus growth *in vitro* [7]. It has also been reported that maoto inhibited influenza virus replication in the lungs of mice and exhibited antipyretic effects [13]. Although no direct inhibition of the growth of influenza viruses by kakkonto has been reported, toll-like receptor 4-dependent adjuvant activity of kakkonto [16] and the possibility of kakkonto inhibiting the onset of influenza encephalopathy by acting on the blood-brain barrier [17] have been reported. Jiinkokato and senkyuchachosan have not been reported to be associated with influenza viruses, and this is the first report about the effects of those Kampo drugs on influenza virus.

A Kampo drug is a mixture of crude drugs derived mainly from plants at a fixed ratio. Maoto and kakkonto both contain the crude drug *Ephedrae herba*. Mantani et al. (1999) showed that *Ephedrae herba* inhibited influenza virus A/PR8 replication in MDCK(+) cells. In this study, maoto was found to be more effective than kakkonto. The ratio of *Ephedrae herba* contained in maoto is about two times larger than that in kakkonto (Table 2), which may indicate that *Ephedrae herba* is a primarily active ingredient. Furthermore, Cinnamomi cortex, a crude drug commonly found in maoto and kakkonto, has also been reported to inhibit virus replication *in vitro* and *in vivo* [18]. This may also be related to the anti-influenza activity.

We studied crude drugs other than *Ephedrae herba* and Cinnamomi cortex in jiinkokato and senkyuchachosan. Anti-influenza virus activity of some of the crude drugs was found. *Glycyrrhizae radix* had a particularly strong anti-influenza virus activity among the crude drugs studied. *Glycyrrhizae radix* has been reported to be effective for a wide range of diseases as an antioxidant, anti-inflammatory, antiviral, and antidiabetic reagent [19]. Its constituents, Isoliquiritigenin and Liquiritigenin, have been reported to have antidepressant, antiparkinsonian, anti-Alzheimer's, and central nervous system-protective effects [20]. The anti-influenza virus activity of *Glycyrrhizae radix* was reported to be effective against influenza virus A/H2N2 in mice infection experiments but not in cultured cells [21]. In this study, we found that *Glycyrrhizae radix* had strong anti-influenza virus activity and that it selectively inhibited influenza virus protein synthesis.

The exact acting point of *Glycyrrhizae radix* is unknown. It was shown in this study that the crude drug did not affect virus particles or increase the pH in endosomes, where influenza virus envelope fusion occurs. The drug thus seems to inhibit viral protein synthesis in the step after viral invasion to a cell. The step may be transport of viral nucleocapsids into the nucleus or viral mRNA synthesis. Polymerase acidic (PA) protein, one of the three polymerases of influenza virus that works for cap snatching and mRNA synthesis, may be the target of *Glycyrrhizae radix*. Shirayama et al. [22] reported that PA endonuclease activity of influenza virus PA protein was inhibited by Kampo drugs *in vitro*. This is the point of action of baloxavir marboxil, an orally administered and long-acting anti-influenza drug. This activity was inhibited by extracts of kakkonto, shosaikoto, saikokeishito, keishito, maobushisaishinto, and maoto but not by an extract of chkujountanto [22]. Two of these Kampo drugs (kakkonto and maoto) were Kampo drugs tested in the present study, and both were effective. *Glycyrrhizae radix* is included in different proportions in all five of the effective Kampo drugs investigated in the present study. The possibility that *Glycyrrhizae radix* inhibits PA nuclease activity and specifically inhibits viral protein synthesis should be investigated in a future study.

Anti-influenza virus activity was not found for bakumondoto, which also contains *Glycyrrhizae radix* (Table 2). Some factors may interfere with the action of *Glycyrrhizae radix* in bakumondoto, or the action of *Glycyrrhizae radix* alone may not be sufficient to work in bakumondoto. Interactions between multiple crude drugs also need to be considered.

In the search for new drugs against influenza viruses, Kampo drugs could be used as seeds for new drugs. For this purpose, it is necessary to analyze crude drugs and identify active ingredients. Drug metabolism and tissue distribution when administered to humans also need to be investigated.

## Figures and Tables

**Table 1:** Kampo drugs investigated in this study.

Chinese characters	Tsumura#	Name (Japanese read)	Name (Chinese read)	Indications
麻黄湯	27	Maoto	Ma-Huang-Tang	Common cold, influenza (in the early stage), rheumatoid arthritis, asthma, nasal obstruction in sucking infants, and suckling difficulty

葛根湯	1	Kakkonto	Ge-Gen-Tang	Common cold, coryza, the initial stage of febrile diseases, inflammatory diseases (conjunctivitis, keratitis, otitis media, tonsillitis, mastitis, and lymphadenitis), shoulder stiffness, neuralgia in the upper body, and urticaria

滋陰降火湯	93	Jiinkokato	Zi-Yin-Jiang-Huo-Tang	Dry cough

川芎茶調散	124	Senkyuchachosan	Chuan-Xiong-Cha-Tiao-San	Common cold, automatic imbalance syndrome peculiar to women resembling climacteric disturbance, and headache

麦門冬湯	29	Bakumondoto	Mai-Men-Dong-Tang	Coughing with a hard, obstructive sputum, bronchitis, and bronchial asthma

**Table 2:** Crude drugs configuring kampo drugs^*∗*^.

#27 maoto	Weight (%)	#1 kakkonto	Weight (%)	#93 jiinkokato	Weight (%)	#124 senkyuchachosan	Weight (%)	#29 bakumondoto	Weight (%)
Armeniacae semen	32.3	Puerariae radix	22.2	*Atractylodis lanceae rhizoma*	13.3	Cyperi rhizoma	20.0	Ophiopogonis radix	37.0
Ephedrae herba	32.3	Zizyphi fructus	16.7	Angelicae acutilobae radix	11.1	*Cnidii rhizoma*	15.0	Oryzae fructus	18.5
Cinnamomi cortex	25.8	Ephedrae herba	16.7	Asparagi radix	11.1	Angelicae dahuricae radix	10.0	Pinelliae tuber	18.5
*Glycyrrhizae radix*	9.7	Cinnamomi cortex	11.1	*Citri unshiu pericarpium*	11.1	Menthae herba	10.0	Zizyphi fructus	11.1
		*Glycyrrhizae radix*	11.1	Ophiopogonis radix	11.1	Notopterygii rhizoma	10.0	Ginseng radix	7.4
		Paeoniae radix	11.1	Paeoniae radix	11.1	*Saposhnikoviae radix*	10.0	*Glycyrrhizae radix*	7.4
		Zingiberis rhizoma	11.1	*Rehmanniae radix*	11.1	Schizonepetae spica	10.0		
				Anemarrhenae rhizoma	6.7	Camelliae sinensis folium	7.5		
				*Glycyrrhizae radix*	6.7	*Glycyrrhizae radix*	7.5		
				Phellodedri cortex	6.7				

^*∗*^Crude drugs investigated in this study are italicized.

## Data Availability

The raw data required to reproduce these findings are available from our laboratory upon request.

## References

[1] Shaw M. L., Palese P., Knipe D. M., Howley P. M. (2013). Orthomyxoviridae. *Fields Virology*.

[2] Thompson W. W. (2003). Mortality associated with influenza and respiratory syncytial virus in the United States. *Jama*.

[3] Poehling K. A., Edwards K. M., Weinberg G. A. (2006). The underrecognized burden of influenza in young children. *New England Journal of Medicine*.

[4] Tumura & Co. (2016). *About Kampo*.

[5] Kubo T., Nishimura H. (2007). Antipyretic effect of Mao-to, a Japanese herbal medicine, for treatment of type A influenza infection in children. *Phytomedicine*.

[6] Toriumi Y., Kamei T., Murata K., Takahashi I., Suzuki N., Mazda O. (2012). Utility of maoto in an influenza season where reduced effectiveness of oseltamivir was observed—a clinical, non-randomized study in children. *Forschende Komplementärmedizin/Research in Complementary Medicine*.

[7] Miyazaki T. (2012). Chinese herbal medicines to inhibit the replication of influenza viruses. *Folia Pharmacologica Japonica*.

[8] Noma K., Kiyotani K., Kouchi H. (1998). Endogenous protease-dependent replication of human influenza viruses in two MDCK cell lines. *Archives of Virology*.

[9] Kawahara T., Akiba I., Sakou M., Sakaguchi T., Taniguchi H. (2018). Inactivation of human and avian influenza viruses by potassium oleate of natural soap component through exothermic interaction. *PLoS One*.

[10] Ueda K., Kawabata R., Irie T., Nakai Y., Tohya Y., Sakaguchi T. (2013). Inactivation of pathogenic viruses by plant-derived tannins: strong effects of extracts from persimmon (Diospyros kaki) on a broad range of viruses. *PLoS One*.

[11] Sakaguchi T., Kiyotani K., Kato A. (1997). Phosphorylation of the Sendai virus M protein is not essential for virus replication either in vitro or in vivo. *Virology*.

[12] Mantani N., Andoh T., Kawamata H., Terasawa K., Ochiai H. (1999). Inhibitory effect of Ephedrae herba, an oriental traditional medicine, on the growth of influenza A/PR/8 virus in MDCK cells. *Antiviral Research*.

[13] Nagai T., Kataoka E., Aoki Y., Hokari R., Kiyohara H., Yamada H. (2014). Alleviative effects of a kampo (a Japanese herbal) medicine “maoto (Ma-Huang-Tang)” on the early phase of influenza virus infection and its possible mode of action. *Evidence-Based Complementary and Alternative Medicine*.

[14] McKimm-Breschkin J. L. (2013). Influenza neuraminidase inhibitors: antiviral action and mechanisms of resistance. *Influenza and Other Respiratory Viruses*.

[15] O’Hanlon R., Shaw M. L. (2019). Baloxavir marboxil: the new influenza drug on the market. *Current Opinion in Virology*.

[16] Ishijima Y., Kawamura T., Kimura A. (2011). Toll-like receptor 4-dependent adjuvant activity of Kakkon-to extract exists in the high molecular weight polysaccharide fraction. *International Journal of Immunopathology and Pharmacology*.

[17] Ohara K., Oshima S., Fukuda N. (2015). The inhibitory effect of kakkonto, Japanese traditional (kampo) medicine, on brain penetration of oseltamivir carboxylate in mice with reduced blood-brain barrier function. *Evidence-Based Complementary and Alternative Medicine*.

[18] Hayashi K., Imanishi N., Kashiwayama Y. (2007). Inhibitory effect of cinnamaldehyde, derived from Cinnamomi cortex, on the growth of influenza A/PR/8 virus in vitro and in vivo. *Antiviral Research*.

[19] Ji S., Li Z., Song W. (2016). Bioactive constituents of Glycyrrhiza uralensis (licorice): discovery of the effective components of a traditional herbal medicine. *Journal of Natural Products*.

[20] Ramalingam M., Kim H., Lee Y., Lee Y.-I. (2018). Phytochemical and pharmacological role of liquiritigenin and Isoliquiritigenin from Radix Glycyrrhizae in human health and disease models. *Frontiers in Aging Neuroscience*.

[21] Kobayashi M., Davis S. M., Utsunomiya T., Pollard R. B., Suzuki F. (1999). Antiviral effect of gingyo-san, a traditional Chinese herbal medicine, on influenza A2 virus infection in mice. *The American Journal of Chinese Medicine*.

[22] Shirayama R., Shoji M., Sriwilaijaroen N., Hiramatsu H., Suzuki Y., Kuzuhara T. (2016). Inhibition of PA endonuclease activity of influenza virus RNA polymerase by Kampo medicines. *Drug Discoveries & Therapeutics*.

